# Design of a polarization-insensitive superconducting nanowire single photon detector with high detection efficiency

**DOI:** 10.1038/srep22710

**Published:** 2016-03-07

**Authors:** Fan Zheng, Ruiying Xu, Guanghao Zhu, Biaobing Jin, Lin Kang, Weiwei Xu, Jian Chen, Peiheng Wu

**Affiliations:** 1 Research Institute of Superconductor Electronics, School of Electronic Science and Engineering, Nanjing University, Nanjing, China 210023

## Abstract

Superconducting nanowire single photon detectors (SNSPDs) deliver superior performance over their competitors in the near-infrared regime. However, these detectors have an intrinsic polarization dependence on the incident wave because of their one-dimensional meander structure. In this paper, we propose an approach to eliminate the polarization sensitivity of SNSPDs by using near-field optics to increase the absorption of SNSPDs under transverse magnetic (TM) illumination. In addition, an optical cavity is added to our SNSPD to obtain nearly perfect absorption of the incident wave. Numerical simulations show that the maximum absorption of a designed SNSPD can reach 96% at 1550 nm, and indicate that the absorption difference between transverse electric (TE) and TM polarization is less than 0.5% across a wavelength window of 300 nm. Our work provides the first demonstration of the possibility of designing a polarization-insensitive and highly efficient SNSPD without performing device symmetry improvements.

Superconducting nanowire single photon detectors (SNSPDs) are a highly attractive candidate in the near-infrared regime because of their superior characteristics, including their high detection efficiency, low dark-count rate, low timing jitter and fast response speed^1^,^2^,^3^,^4^,^5^,^6^,^7^,^8^,^9^,^10^,^11^,^12^. Because of the nanowire meander structure of SNSPDs, the absorption characteristics of the SNSPDs show an intrinsic sensitivity to the polarization states of incident waves^13^,^14^, which can lead to restrictions in certain applications. One such example is the quantum key distribution system based on the differential phase shifted keying method^15^, in which all of the information is encoded into the phase difference between two nearby photons. Because the key information is unrelated to the photon polarization state, which may fluctuate during the process of fibre-optic transmissions, using an SNSPD with polarization sensitivity will result in an increased bit-error-rate of the transmitted keys, which is not desirable.

It is well-known that the system efficiency *η*_system_ of the SNSPD can be expressed as follows^16^:


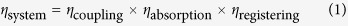


where *η*_coupling_ is the device coupling efficiency, *η*_absorption_ is the device absorption efficiency, and *η*_registering_ is the device intrinsic efficiency. Generally, *η*_coupling_ and *η*_registering_ are considered to be insensitive to polarization, and *η*_registering_ is considered to be the major cause of SNSPD polarization dependence^16^. The most straightforward method of removing the absorption polarization sensitivity is to improve the symmetry property of the device structure. To this end, Dorenbos *et al.* have demonstrated a polarization-insensitive SNSPD by changing the meander line structure into a spiral line structure^17^; however, this change reduced the device performance because an intermediate absorption efficiency had to be accepted. Verma *et al.* have demonstrated a polarization-insensitive SNSPD by using a dual-layer WSi meander structure with perpendicular meander orientations^18^. However, from a lattice matching perspective, it is difficult to fabricate the dual-layer structure using NbN film, which is used more frequently than WSi film in SNSPDs because of its high critical temperature advantage.

Here, we present the design of a polarization-insensitive SNSPD with high detection efficiency based on NbN films. Compared with methodologies in previous studies, our design methodology does not involve any improvements to the geometric symmetry of the SNSPD. Instead, we first use classical electromagnetic wave theory to analyse the near-field features of the electric field inside the NbN nanowire and reveal the origin of the absorption polarization sensitivity of the superconducting nanowire meander structure. Based on our findings, we then propose a high-index dielectric material based compensation method to mitigate the polarization dependence of SNSPDs. We also adopt the concept of critical coupling^19^ to further optimize the performance of the SNSPD by embedding it into a single-port cavity. Finally, to validate our design method, we design a polarization-insensitive and highly efficient SNSPD that operates in the 1550 nm region. Numerical simulations show that the maximum absorption of the designed SNSPD is 96% and the absorption difference between two polarization states is less than 0.5% across a wavelength window of 300 nm.

## Results

### Origin of the polarization dependence of SNSPD

The superconducting nanowire of a SNSPD is normally fabricated in a meander pattern, which can be approximated as a 1D periodic structure. The optical absorption (denoted by A) of such a periodic structure is as follows^13^:


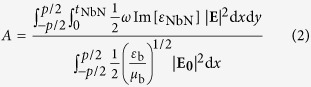


where **E**_0_ denotes the incident electric field; **E** denotes the electric field inside the superconducting nanowire; p denotes the pitch of the unit cell; *ε*_NbN_ and *t*_NbN_ denote the permittivity and thickness of the superconducting nanowire, respectively; and ε_b_ and μ_b_ denote the permittivity and permeability of the background material, respectively. It is apparent from equation (2) that when the incident field **E**_0_ is given, the absorption A is determined by the internal field **E**. Therefore, investigating the near-field features of **E** within the nanowire cross-section is of great importance for determining the origin of the polarization sensitivity of SNSPDs.

**Figure 1:**
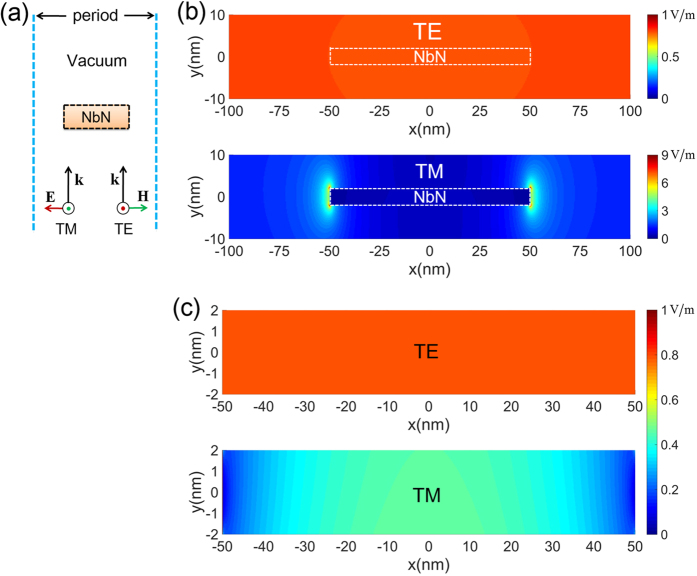
Polarization dependent optical response of a NbN nanowire. (**a**) Unit cell for the NbN nanowire meander placed in a vacuum. (**b**) The distribution of the electric field amplitude around (**b**) and within (**c**) the nanowire for cases of TE and TM illumination. The excitation wavelength is at 1550 nm.

We first study the electromagnetic responses of a 1D superconducting nanowire meander under illumination with two orthogonal polarizations. As shown in Fig. 1(a), the unit cell consists of a NbN nanowire that is placed in a vacuum and has a width and thickness of 100 nm and 4 nm, respectively. The unit cell has a pitch of 200 nm, and it is excited by an optical plane wave incident from its bottom boundary. The amplitude of the incident electric field is 1 V/m, and the wavelength of the incident wave is 1550 nm. Using a commercially available optical wave simulation software program (see the Methods section for details), the distribution of the amplitude of the scattered overall electric field is numerically calculated. The results are shown in Fig. 1(b) for positions around the NbN nanowire and in Fig. 1(c) for positions inside the NbN nanowire. When the unit cell is illuminated by transverse electric (TE) polarized light (wherein the electric field is parallel to the superconducting nanowire), the electric field is uniformly distributed within and outside the NbN nanowire and has an amplitude of ~0.8 V/m. However, when the unit cell is illuminated by transverse magnetic (TM) polarized light (wherein the electric field is perpendicular to the superconducting nanowire), we find that for positions outside the NbN nanowire, the electric fields are strongly enhanced, particularly around the four corners. Most importantly, we note that for positions within the NbN nanowire, two low electric field intensity regions occur close to the left and right boundaries of the NbN nanowire, where the electric field amplitude gradually increases from ~0.1 V/m (measured at the boundaries of the nanowire) to ~0.4 V/m (measured close to the centre of the nanowire). The presence of these two low electric field intensity regions for case of TM polarization clearly explains the polarization sensitivity of a regular SNSPD. Thus, any effort to eliminate the low electric field intensity regions could help to relieve the SNSPD polarization problem.

### Eliminating the low electric field intensity regions

**Figure 2:**
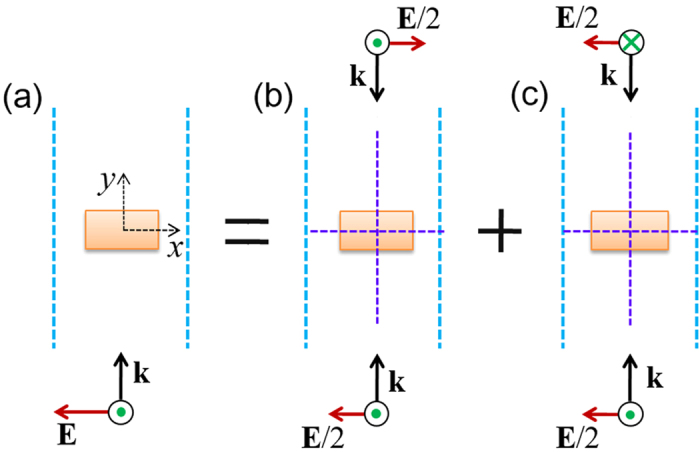
Even-odd mode analysis of the optical response of a NbN nanowire under TM illumination. The realistic excitation (**a**) is decomposed as a superposition of an odd virtual excitation (**b**) and an even virtual excitation (**c**).

We aim to identify an approach for eliminating the low electric field intensity regions associated with TM polarized light. We first use the classical electromagnetic wave theory to analyse the near-field features within the NbN nanowire under TM illumination. Because of the symmetry of the unit cell presented in Fig. 1(a), the method of even-odd mode analysis is adopted here^19^. In Fig. 2, we illustrate a decomposition of the realistic excitation as a superposition of two virtual excitations. For the first virtual excitation [Fig. 2(b)], the electric field of the two paired sources possesses odd symmetry with respect to the y = 0 plane (dashed horizontal purple line), whereas for the second virtual excitation [Fig. 2(c)], the electric field of the two paired sources possesses even symmetry with respect to the y = 0 plane (dashed horizontal purple line). Because the overall electric field of the odd excitation [Fig. 2(b)] is zero at the centre plane (y = 0) of the nanowire and the thickness of the nanowire is much smaller than the excitation wavelength, the optical response of the NbN nanowire under odd excitation is negligible, indicating that the major role of nanowire absorption occurs in the even excitation mode.

For the even excitation shown in Fig. 2(c), the electric field components (according to Maxwell’s equations) read as follows:


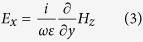



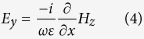


**Figure 3:**
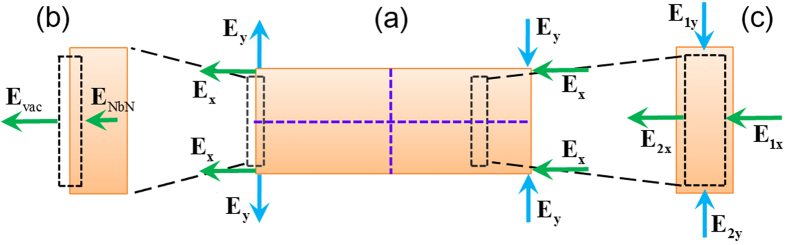
Near field analysis on the origin of the polarization dependent absorption of the NbN nanowire. (**a**) Illustration of the symmetry properties of the electric field components under TM illumination. (**b**) A micro-unit taken from the left boundary of the nanowire. (**c**) A micro-unit taken from an arbitrary position inside the nanowire.

Figure 2(c) shows that the magnetic field H_z_ possesses an even symmetry with respect to the x = 0 plane (dashed vertical purple line) and an odd symmetry with respect to the y = 0 plane (dashed horizontal purple line). Because the symmetry properties will flip after derivatives are taken, equations (3) and (4) indicate that under cases of even excitation, E_x_ possesses even symmetries with respect to both the x = 0 plane and y = 0 plane, and E_y_ possesses odd symmetries with respect to both the x = 0 plane and y = 0 plane. The symmetry properties of the electric fields under TM illumination are graphically summarized in Fig. 3(a).

Since E_y_ possesses old symmetry, its amplitude is zero along the two symmetry planes located at x = 0 and y = 0. As a result, E_y_ is mainly confined around the four corners of the nanowire. On the other hand, E_x_ distributes uniformly within the nanowire due to the lack of nulls (even symmetry). Because of such features, the integral result in equation (2) contributed by the E_y_ component is much smaller than that contributed by the E_x_ component (note that this conclusion has been confirmed numerically). In other words, under TM illumination, the nanowire absorption is primarily caused by the E_x_ component. Thus, it follows that to enhance the absorption for cases of TM polarization, we only need to eliminate the low electric field intensity regions associated with E_x_, which can be performed by: (1) increasing the boundary values of E_x_ at the left and right sides of the NbN nanowire where the low electric field intensity regions begin, and (2) reducing the width of the low electric field intensity regions by increasing the slope rate of E_x_ inside the NbN nanowire.

To analyse the boundary values of E_x_, consider a micro-unit taken from the left boundary of the NbN nanowire. As shown in Fig. 3(b), the boundary condition of Maxwell’s equations indicate the following:


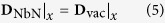


With modifications, we obtain


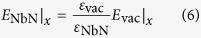


Because the permittivity constant of the vacuum has a much smaller modulus than that of the NbN (~60 at 1550 nm^13^,^20^), it follows from equation (6) that the boundary value of the amplitude of E_x_ inside the NbN nanowire is greatly suppressed. Therefore, if we fill the spaces at the left and right sides of the NbN nanowire with dielectric materials that have large permittivity, the boundary value of E_x_ at the left and right sides of the NbN nanowire might be increased.

To analyse the slope rate of E_x_, consider a micro-unit taken from an arbitrary position inside the NbN nanowire. As shown in Fig. 3(c), due to the lack of free charges inside the NbN nanowire, we have ∯  **D**·ds = 0, where the integral represents the flux of the displacement vector over the boundary of the micro-unit. Note that the NbN permittivity inside the nanowire is a spatially independent constant, the flux integral is then reduced to ∯  **E**·ds = 0. Since E_y_ possesses an odd symmetry with respect to the y = 0 plane, for E_y_ located at the bottom and upper boundaries of the micro-unit, we have E_1y_ = −E_2y_. On the other hand, because E_x_ possesses an even symmetry with respect to the y = 0 plane and the thickness of the NbN nanowire is much smaller than the excitation wavelength, E_x_ along the left and right boundaries of the micro-unit can be approximated as a constant and taken out of the flux integral. It follows that the electric field flux integral over the boundary of the micro-unit then reads as follows:


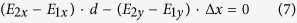


where d and ∆x denote the height and the width of the micro-unit respectively. With modifications, we obtain


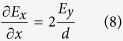


Equation (8) shows that the slope rate of E_x_ along the x direction is proportional to E_y_. Therefore, to increase the slope rate of E_x_ (for purpose of reducing the width of the low electric field intensity regions), inside the NbN nanowire E_y_ must be enhanced. Following a boundary value analysis of E_y_, which is similar to that illustrated by equations (5) and (6), we conclude that if the spaces at the bottom and upper sides of the NbN nanowire are filled with dielectric materials that have large permittivity, the amplitude of E_y_ within the NbN nanowire might be increased, leading to an enhanced slope rate of E_x_ and thus a reduced width of the low electric field intensity regions.

**Figure 4:**
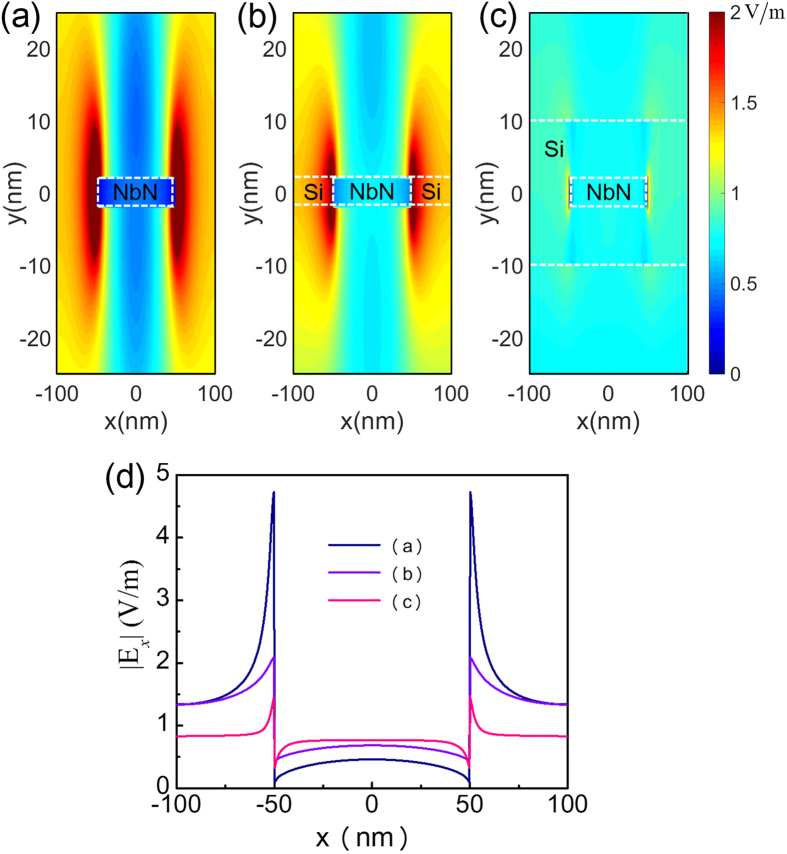
High index dielectric material based compensation of the polarization dependent absorption of the NbN nanowire. Distribution of the electric field amplitude for cases in which the NbN nanowire is placed in a vacuum (**a**), the NbN nanowire has left- and right-side spaces filled with Si (**b**), the NbN nanowire has a 20-nm thick wrapping layer composed of Si (**c**). The distribution of the amplitude of E_x_ is along the middle line (y = 0) of the NbN nanowire (**d**).

We use numerical simulations to validate the above conclusions on the boundary value and slope rate of E_x_. In our simulations, we choose Si as the compensation material because its permittivity is approximately 12 at 1550 nm^29^. Figure 4(a–c) show the distribution of the electric field amplitude for cases under TM illumination where the NbN nanowire is placed in a vacuum, the NbN nanowire has left- and right-side spaces filled with Si, and the NbN nanowire has a 20 nm thick wrapping layer composed of Si, respectively. To facilitate the comparison, Fig. 4(d) summarizes the distributions of the amplitude of E_x_ along the middle plane (y = 0) of the NbN nanowire for the three cases. Compared with the case in which the NbN nanowire is placed in a vacuum, Fig. 4(d) shows that when Si is used to fill the spaces to the left and right of the nanowire, the electric field amplitude at the inner sides of the NbN boundaries is significantly increased, although its slope rate remains almost unchanged. When the nanowire is wrapped by a Si layer, the boundary value and the slope rate of the electric field are significantly enhanced. Our electromagnetic wave analysis of the low electric field intensity regions of the NbN nanowire for the case of TM illumination is therefore completely supported by the numerical simulations.

### Numerical design of the polarization-insensitive and highly efficient SNSPD

The above analysis illustrates a high-index dielectric material based method of eliminating the polarization dependence of SNSPDs by improving their absorption under TM illumination. In this section, we combine this method with the concept of critical coupling to design a polarization-insensitive SNSPD with high detection efficiency. Note that the central idea is to place the NbN nanowire wrapped by a Si layer into a single-port resonator, adjust the thickness of the Si wrapping layer to remove polarization sensitivity, and adjust other resonator parameters to optimize the absorption curves. Subsequently, we will follow this idea to design a polarization-insensitive and highly efficient SNSPD that operates in the 1550 nm region. In our design, the thickness and width of the NbN nanowire are 4 nm and 100 nm, respectively. The remaining design parameters are determined from numerical simulations (see the Methods section for details).

**Figure 5:**
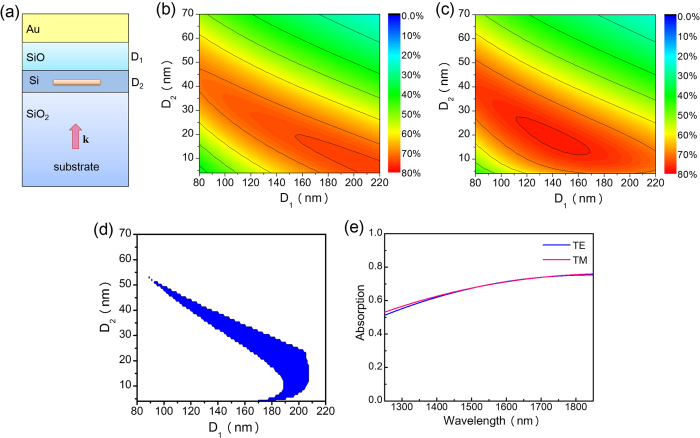
Results for the first step of the design process. (**a**) Schematics of the unit cell. (**b**) Contour plots of the absorption as a function of D_1_ and D_2_ under cases of TE (**b**) and TM (**c**) illumination. The excitation wavelength is at 1550 nm. (**d**) Absorption phase diagram derived from (**b**,**c**). The blue region denotes the parameter range within which the absorption for TE and TM cases is larger than 65%, and the difference between these two cases is less than 1%. (**e**) Absorption curves for cases of TE and TM polarization. In (**a**), D_1_ denotes the thickness of the upper cavity layer composed of SiO, and D_2_ denotes the thickness of the warping layer composed of Si.

We start the design process by considering the unit cell shown in Fig. 5(a). The NbN nanowire is wrapped by a Si layer (with a thickness denoted by D_2_) and then placed into an open cavity^21^ consisting of a substrate composed of SiO_2_, an upper cavity layer composed of SiO (with a thickness denoted by D_1_) and a reflection layer composed of Au (with a thickness of 400 nm). Note that D_1_ (upper cavity layer) increases the absorption by introducing a quarter-wavelength resonance^21^, and D_2_ (Si wrapping layer) removes the absorption sensitivity. We use numerical simulations to determine the optimized values of D_1_ and D_2_. Figure 5(b,c) depict the contour plots of the absorption as functions of D_1_ and D_2_ for cases of TE and TM polarization respectively. To generate these plots, the excitation wavelength is fixed at 1550 nm. From Fig. 5(b,c), an absorption phase diagram can be created, and the result is shown in Fig. 5(d), where the blue region denotes the parameter range within which the absorption for the TE and TM cases is larger than 65%, and the difference between these two cases is less than 1%. This region is suitable for the design of a polarization-insensitive SNSPD with high detection efficiency. From Fig. 5(d), we select D_1_ = 180 nm and D_2_ = 25 nm because at this point, the blue region has a wide opening, which relaxes the fabrication tolerance. Figure 5(e) depicts the absorption curves calculated within a wavelength window of 1250 nm–1850 nm under cases of TE and TM illumination. By wrapping the NbN nanowire with a Si layer, the polarization sensitivity of the SNSPD is effectively removed. However, the maximum absorption is less than 70%, indicating that further improvements can be made.

**Figure 6:**
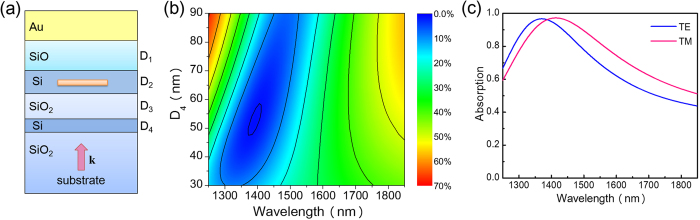
Results for the second step of the design process. (**a**) Schematics of the unit cell. (**b**) Contour plot of the reflectance as a function of D_4_ and the excitation wavelength, with values averaged between cases of TE and TM polarization. (**c**) Absorption curves for cases of TE and TM polarization. In (**a**), D_3_ denotes the thickness of the lower cavity layer composed of SiO_2_, and D_4_ denotes the thickness of the partially reflective mirror layer composed of Si.

To increase the absorption, we close the open cavity by adding a partially reflective mirror and then exploit the concept of critical coupling to achieve maximum absorption^19^. As shown in Fig. 6(a), a second Si layer is inserted underneath the NbN nanowire to work as a partially reflective mirror of the closed cavity. We denote the thickness of the lower cavity layer by D_3_ and the thickness of the partially reflective mirror layer by D_4_. According to the concept of critical coupling, for a single-port resonator, if the coupling loss is adjusted to be equal to the absorption loss, the incident wave will be totally absorbed, resulting in a zero reflectance. Therefore, by adjusting D_4_ (thickness of the partially reflective mirror layer), the absorption can be increased to ~100%. Figure 6(b) depicts the reflectance as a function of D_4_ and the incident wavelength. To generate this plot, we set the thickness of the lower cavity layer to be equal to that of the upper cavity layer, i.e., D_3_ = D_1_ = 180 nm, because these two layers have similar refractive indexes. Moreover, we set the polarization angle of the electric field to 45° with respect to the nanowire plane. As a result, the obtained reflectance is an average between cases of TE and TM polarization. Figure 6(b) shows that when D_4_ = 52 nm and λ = 1390 nm, the averaged reflectance is minimized (0.87%). The corresponding absorption curves for the TE and TM polarization are illustrated in Fig. 6(c). Although the peak absorption for the TE and TM polarization reaches 96% (note that the other 4% is absorbed by the top Au reflector), the two peak absorption wavelengths are not aligned and deviate from the desired wavelength, which is 1550 nm.

**Figure 7:**
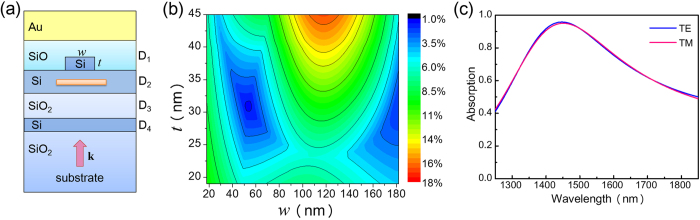
Results for the third step of the design process. (**a**) Schematics of the unit cell. (**b**) Contour plot of the maximum value of the absorption difference between cases of TE and TM polarization as a function of w and t. Each point of the contour plot is obtained by scanning the wavelength from 1250 nm to 1850 nm. (**c**) Absorption curves for cases of TE and TM polarization. In (**a**), w and t denote the width and thickness of the birefringence compensation nanowire composed of Si.

The discrepancy between the two peak absorption wavelengths is caused by the birefringence effect of the NbN nanowire, which can be compensated for by adopting the technique of resonator perturbations^19^. According to the resonator perturbation theory, if the dielectric material of a resonator is perturbed by an amount denoted by Δε, then the resonant frequency will be changed by an amount that is proportional to Δε. Consequently, because the real portion of the permittivity of the NbN nanowire is negative (since the wire is metallic), if we add into the cavity a dielectric material nanowire for which the real portion of the permittivity is positive, then it is possible to compensate for the birefringence effect caused by the NbN nanowire. As shown in Fig. 7(a), a birefringence compensation nanowire composed of Si is added to the cavity, and its width and thickness are denoted by w and t, respectively. Figure 7(b) illustrates the maximum value of the absorption difference between cases of TE and TM illumination as a function of w and t, which is obtained by scanning the wavelength from 1250 nm to 1850 nm. Figure 7(b) shows that when w = 54 nm and t = 31 nm, the difference between the two absorptions is minimized. The corresponding absorption curves are illustrated in Fig. 7(c), which shows that although the absorption curves for cases of TE and TM polarization are perfectly aligned, their absorption peak wavelengths deviate from the desired wavelength, which is 1550 nm.

**Figure 8:**
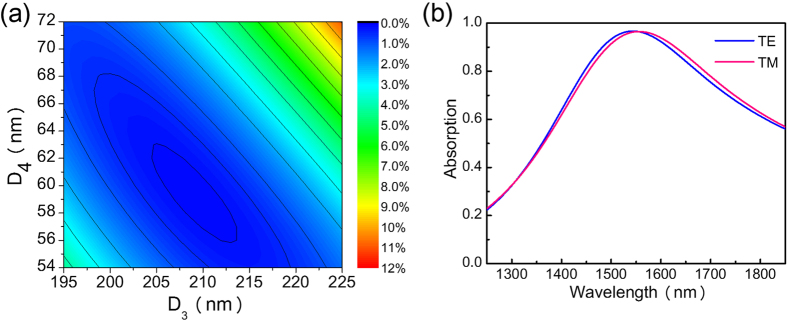
Results for the fourth step of the design process. (**a**) Contour plot of the reflectance as a function of D_3_ and D_4_ at 1550 nm, with values averaged between cases of TE and TM polarization. (**b**) Absorption curves for cases of TE and TM polarization.

The peak absorption wavelength can be shifted to 1550 nm by adjusting D_3_ and D_4_ (thicknesses of the lower cavity layer and the partially reflective mirror layer, respectively), which control the round trip phase of the cavity. Figure 8(a) depicts the reflectance as a function of D_3_ and D_4_ and the excitation wavelength is fixed at 1550 nm. To generate this plot, we set the polarization angle of the electric field to 45° with respect to the nanowire plane. As a result, the obtained reflectance is an average between cases of TE and TM polarization. Figure 8(a) shows that when D_3_ = 209 nm and D_4_ = 59 nm, the averaged reflectance is minimized (0.14%). The corresponding absorption curves are displayed in Fig. 8(b). Although the peak absorption wavelengths for cases of TE and TM polarization are shifted to 1550 nm, a slight wavelength walk-off between the two absorption curves can be observed.

**Figure 9:**
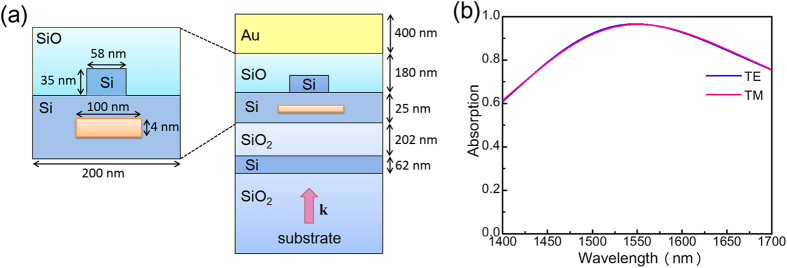
Final results for the polarization-insensitive and highly efficient SNSPD designed after iterations. (**a**) Schematics of the unit cell. (**b**) Absorption curves for cases of TE and TM polarization.

A comparison of Fig. 8(b) with Fig. 6(c) shows that the performance of the SNSPD is significantly improved. To further optimize the design result, we iterate the design process by repeating the steps associated with Figs 7 and 8. The final values of the design parameters are summarized in Fig. 9(a), and the corresponding absorption curves for cases of TE and TM illumination are shown in Fig. 9(b). By applying the iterations, the two absorption curves overlap well with each other; the absorption peak is positioned at 1550 nm; the maximum absorption of the SNSPD is 96%; and the absorption difference is less than 0.5% across a wavelength window of 300 nm.

**Figure 10:**
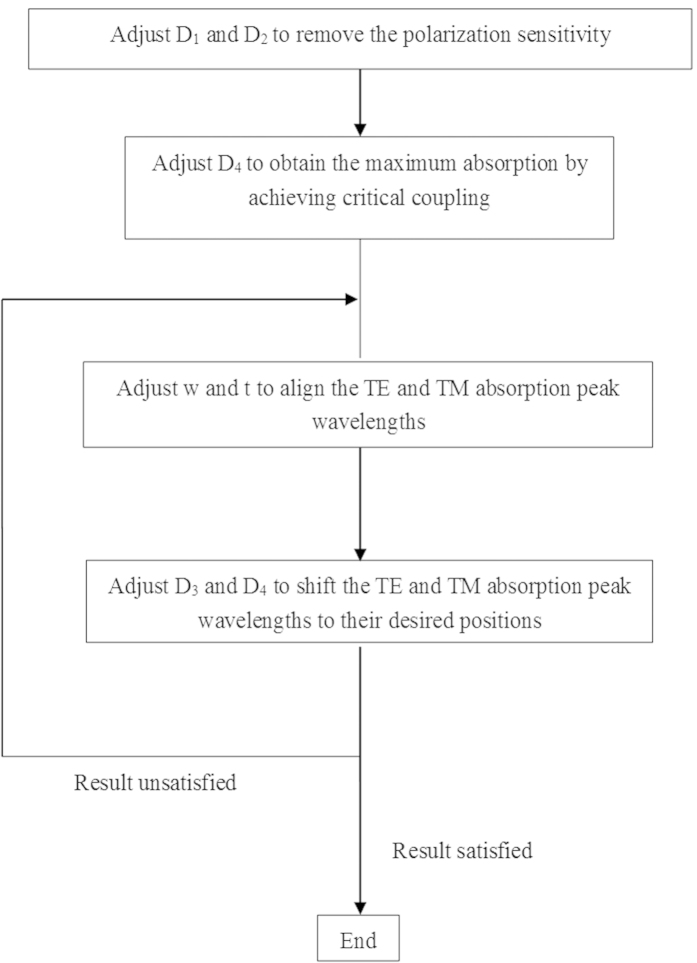
Flowchart for the design of a polarization-insensitive SNSPD with high detection efficiency. See Fig. 7(a) for parameter definitions.

We conclude this section by summarizing the above design process using a flowchart, which is shown in Fig. 10, to facilitate the design of polarization-insensitive SNSPDs with high detection efficiency.

## Discussion

**Figure 11:**
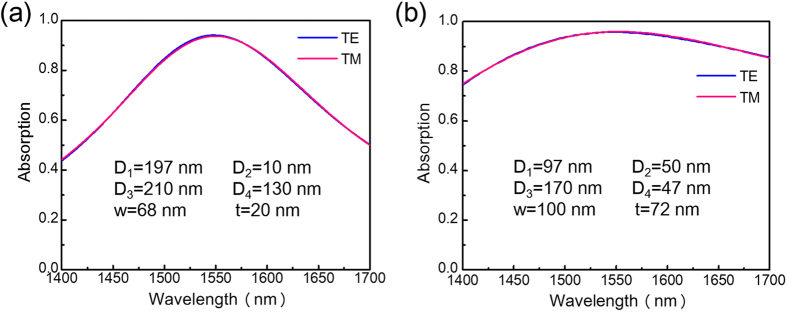
Effects on the variation of NbN permittivity at low temperature. Absorption curves for cases of TE and TM polarization, showing the effect that the room temperature based Drude model NbN permittivity is divided by 2 (**a**) and multiplied by 2 (**b**). The SNSPD design parameters are listed in the figures.

In our simulations, we used a room temperature-based Drude model for the permittivity of NbN (see the Methods section for details). Although it has been noted that the permittivity of NbN should not change substantially between low and high temperatures^22^, it is still of interest to investigate the effect of NbN permittivity deviations on the performance of the designed devices. Note that at room temperature, the modulus of the NbN permittivity is ~60 at 1550 nm^13^,^20^. We assume that at low temperatures, the Drude model-based NbN permittivity is either reduced by a factor of 2 (yielding a modulus of ~30 at 1550 nm) or increased by a factor of 2 (yielding a modulus of ~120 at 1550 nm). Using these two modified NbN permittivities, the SNSPDs are redesigned. The corresponding results for the absorption curves are plotted in Fig. 11(a) for cases reduced by a factor of 2 and in Fig. 11(b) for cases increased by a factor of 2. Our results show that even when there is a change in NbN permittivity by a factor of 4, with the help of our methodology, the SNSPDs can still be designed to possess nearly ideal polarization insensitivity and high efficiency. Moreover, from Figs 8 and 11, we observe that when the modulus of the NbN permittivity is increased, the thickness of the Si warping layer and the area of the Si compensation nanowire are increased, the thickness of the Si partially reflective mirror layer is decreased, and the spectral width of the absorption curves is increased. These observations are in agreement with our near field optics analysis and the theory of critical coupling. Similarly, note that we also investigated the effect of Au permittivity low-temperature deviations on the performance of the designed devices. Our results show that when the Au permittivity is either reduced or increased by a factor of 2 at low temperature, except a slight shift of the peak absorption wavelength, the performance of the devices is unaffected, even if the SNSPDs are not redesigned.

In summary, the design of a polarization-insensitive SNSPD with high detection efficiency has been presented. Through a detailed electromagnetic wave analysis that focuses on the distribution of the electric field inside the nanowire, we developed a high-index dielectric material based compensation method to improve the absorption of the NbN nanowire under case of TM illumination. Using the flowchart proposed in this article, we designed a polarization-insensitive and highly efficient SNSPD operating in the 1550 nm region. Numerical simulations show that the maximum absorption of the designed SNSPD is 96% and the absorption difference between cases of TE and TM polarization is less than 0.5% across a wavelength window of 300 nm. It is shown that our design methodology is effective for a broad range of NbN permittivities.

## Methods

The numerical simulations were performed in a 2D manner with a software based on the finite-difference time-domain method (FDTD Solutions, Lumerical Inc.). The computer used for this research is a personal computer with an Intel Core™-I7-4790 CPU and 64 GB RAM. Because of the quasi-periodic condition of the NbN meander, the simulated object is a unit cell that has periodic boundary conditions on its left and right boundaries and perfect matched layer conditions at its bottom and top boundaries. The thickness and width of the NbN nanowire are 4 nm and 100 nm, respectively, and the pitch of the unit cell is 200 nm. Note that in our simulations, for near field positions inside the NbN nanowire, the Si nanowire and the Si wrapping layer, the mesh size is 0.5 nm. At other far field positions, the minimum and maximum mesh sizes are 0.5 nm and 50 nm, respectively.

The literature has indicated that the permittivity of NbN is treated by a Drude model^13^,^20^. Although this Drude model is derived from measurements performed at room temperature^13^, it was noted that the permittivity of NbN should not change substantially between low and high temperatures if the photon energy (~800 meV at 1550 nm) is much larger than the energy gap (~1 meV) of NbN^22^. Accordingly, various groups from institutes such as the NIST and MIT have used the room temperature based Drude model to design SNSPDs^13^,^20^,^24^,^25^,^26^,^27^,^28^. Following these groups, such a Drude model is also adopted in this study. The nonlinear effects associated with NbN are not considered because the optical power is at the level of single photons.

Additional design parameters are indicated below. The permittivity of Au is treated with a combined Lorentz and Drude model^23^. The permittivity of SiO, SiO_2_ and Si are n_SiO_ = 1.551, n_SiO2_ = 1.444 and n_Si_ = 3.497, respectively^29^. Note that we have included the same assumptions outlined in ref. 22 and considered that these room temperature based permittivities can be used at temperatures below the critical temperature of NbN (~10 K). Our treatment is justified because the measured data have been shown to be consistent with the simulated data for a designed and fabricated SNSPD that has an Au layer as the top reflection mirror^13^.

## Additional Information

**How to cite this article**: Zheng, F. *et al.* Design of a polarization-insensitive superconducting nanowire single photon detector with high detection efficiency. *Sci. Rep.* **6**, 22710; doi: 10.1038/srep22710 (2016).
